# On the sensitivity of steady-state free precession myocardial blood-oxygen-level-dependent MRI at 1.5 T: theory and experiment

**DOI:** 10.1186/1532-429X-11-S1-O82

**Published:** 2009-01-28

**Authors:** Xiangzhi Zhou, Richard Tang, Rachel Klein, Debiao Li, Rohan Dharmakumar

**Affiliations:** 1 Department of Radiology, Northwestern University, Chicago, IL USA; 2 Northwestern University, Chicago, IL USA

**Keywords:** Flip Angle, Main Magnetic Field, Susceptibility Difference, Blood Volume Fraction, Vessel Axis

## Introduction

Through theoretical simulations and experimental studies, it has been shown in whole blood and in skeletal muscle that SSFP-based BOLD contrast is strongly dependent on repetition time(TR) and flip angle(FA). While these studies accounted for spin exchange effects between intra-(IV) and extra-vascular(EV) spaces, they have largely ignored the diffusion effects, particularly in the EV space. Since most of the blood present in the microvasculature of the heart is in the capillaries, we hypothesize that diffusion-mediated effects is an important mechanism allowing for the detection of oxygenation changes with SSFP-based myocardial BOLD imaging.

## Purpose

To investigate the effect of TR and FA on SSFP-based myocardial BOLD sensitivity through a theoretical model accounting for diffusion effects and validate it using a controlled canine model.

## Methods

### Monte-Carlo simulations(MCS)

Approximating capillaries as an infinite cylinder, the magnetic field variation (ΔB_z_) outside the capillary vessel was computed as B_0_Δχ(R/r)^2^ cos(2φ)sin^2^θ/2 (EV) and B_0_Δχ(cos^2^θ-1/3)/2 (IV), where R is the vessel radius, r the distance from vessel axis, θ the angle between the main magnetic field (B_0_) and the vessel axis, and φ the angle between r and the projection of the main magnetic field onto the plane orthogonal to the vessel axis. The susceptibility difference Δχ between IV and EV space is: Δχ = Hct(1-Y)ΔX, where ΔX = 3.39 ppm is the susceptibility difference between fully oxygenated and deoxygenated hemoglobin, Hct is hematocrit(0.4), and Y is oxygen saturation(20% and 80%). Spin diffusion was modeled as 3D Brownian motion of 5000 spins inside a cubic box with time steps of 50 μs and diffusion coefficient of 1.5 × 10^-9^m^2^/s. Other parameters were: R = 6 × 10^-6^m, blood volume fractions = 9%, number pulses to reach steady-state = 2000; TR = 3.5, 4.7, 6.0 ms; and FA= 30°, 50°, 70°.

### Experimental studies

An external hydraulic occluder was placed around the left circumflex coronary(LCX) for the purpose of inducing reversible stenosis in 4 dogs. Following recovery(1 week), animals were sedated, ventilated and placed in the scanner (1.5 T Siemens Espree). ECG-gated and multiple breath-held 2D-Cine SSFP sequences were prescribed under pharmacological stress with and without LCX stenosis over the LV. Three short-axis images with centre slice located on mid-LV were acquired for each study. Scan parameters: in-plane resolution = 1.2 × 1.2 mm^2^, slice thickness = 5 mm, TR/TE = 6.0 ms/3.0 ms, 4.7 ms/2.35 ms, 3.5 ms/1.75 ms, flip angle = 70°, 50°, 30°, segments/cardiac phase were adjusted to achieve an optimal temporal resolution(10 ms–20 ms) that minimize motion/flow artifacts.

### Data analysis

Regional SSFP BOLD signal contrast, [I_LAD_-I_LCX_]/I_LAD_, was used to evaluated the SSFP BOLD sensitivity, where I_LAD_ and I_LCX_ are average SSFP signal intensities measured within LAD and LCX territories.

## Results

**Figure 1:**
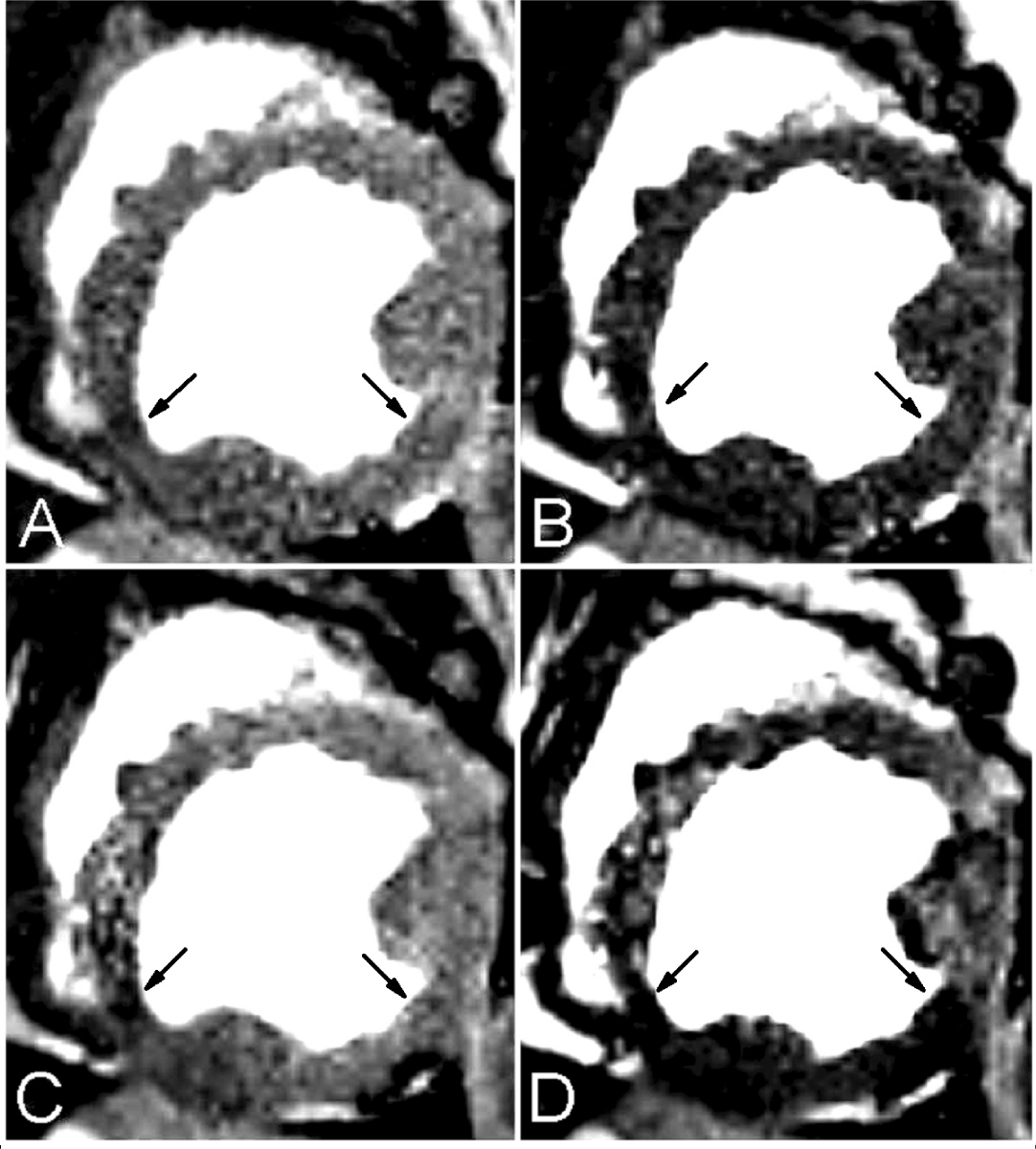
**2D late diastole cine SSFP BOLD images of a dog with severe LCX stenosis and adenosine infusion obtained at different TR and FA: TR/FA = 3.5 ms, 30° (A); 3.5 ms, 70° (B); 6.0 ms, 30° (C); and 6.0 ms, 70° (D)**. LCX territories are the shorter arcs subtended by arrows.

**Figure 2:**
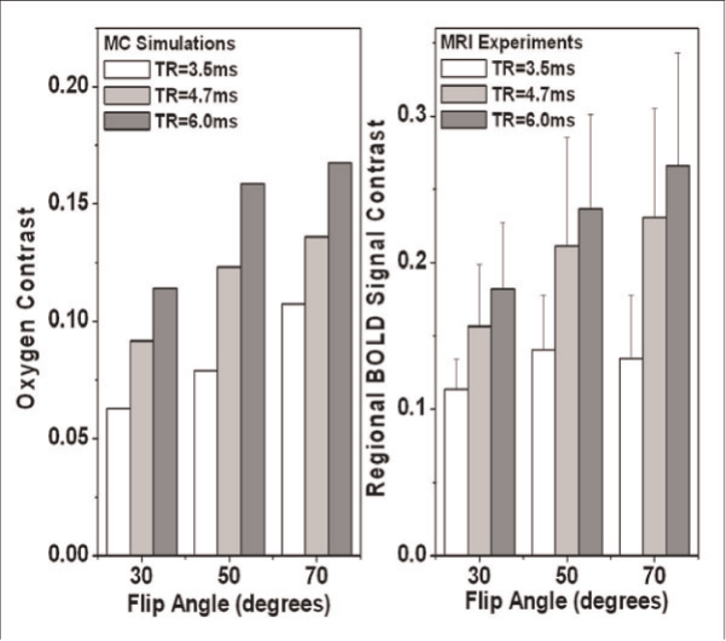
**SSFP-baseed Myocardial BOLD contrast obtained from MCS and experimental studies with the same parameters (TR = 3.5 ms, 4.7 ms, 6.0 ms; Flip angle = 30°, 50°, 70°)**. For a given FA and TR, MCS oxygen contrasts were computed assuming myocardial oxygenation in the territory supplied by a stenotic vessl is 20%, while that supplied by a healthy vessel is 80% under pharmacological stress, assuming myocardial blood volume fraction of 9%.

Figure 1 shows a typical set of 2D late diastole short-axis cardiac images from SSFP cine scans of a dog with severe stenosis and adenosine infusion for different TR and FA. Note that the global and regional signal intensities are strongly dependent on imaging parameters. Figure 2 shows the SSFP BOLD signal contrast obtained from MCS and experimental studies. The regional BOLD signal contrast between LAD and LCX supplying regions match well with the MCS.

## Discussion

MCS and experimental studies showed that TR and FA play a significant role in determining SSFP-based myocardial BOLD contrast. In particular, both MCS and experiments showed that increasing the FA or TR gave a concomitant increase in SSFP-based myocardial BOLD contrast. We found that a combination of TR/FA = 6.0 ms/70° gave the highest oxygen contrast among all the parameter sets studied, although more artifacts were observed in certain cardiac phases as TR was increased from 3.5 ms. In order to enable the entire cine image set to provide reliable regional BOLD contrast at large TRs, robust artifact correction methods need to be developed.

